# Gray Matter Matters: A Longitudinal Magnetic Resonance Voxel-Based Morphometry Study of Primary Progressive Multiple Sclerosis

**DOI:** 10.3389/fneur.2020.581537

**Published:** 2020-11-12

**Authors:** Ted L. Rothstein

**Affiliations:** Department of Neurology, Multiple Sclerosis Clinical Care and Research Center, George Washington University School of Medicine, Washington, DC, United States

**Keywords:** primary progressive multiple sclerosis, voxel based morphometry, MR imaging, gray matter, memory deficit

## Abstract

**Background:** Multiple Sclerosis (MS) lesions in white matter (WM) are easily detected with conventional MRI which induce inflammation thereby generating contrast. WM lesions do not consistently explain the extent of clinical disability, cognitive impairment, or the source of an exacerbation. Gray matter (GM) structures including the cerebral cortex and various deep nuclei are known to be affected early in Primary Progressive Multiple Sclerosis (PPMS) and drive disease progression, disability, fatigue, and cognitive dysfunction. However, little is known about how rapidly GM lesions develop and accumulate over time.

**Objective:** The purpose of this study is to analyze the *degree* and *rate of progression* in 25 patients with PPMS using voxel-based automated volumetric quantitation.

**Methods:** This is a retrospective single-center study which includes a cohort of 25 patients with PPMS scanned utilizing NeuroQuant® 3 dimensional voxel-based morphometry (3D VBM) automated analysis and database and restudied after a period of ~1 year (11–14 months). Comparisons with normative data were acquired for whole brain, forebrain parenchyma, cortical GM, hippocampus, thalamus, superior and inferior lateral ventricles. GM volume changes were correlated with their clinical motor and cognitive scores using Extended Disability Status Scales (EDSS) and Montreal Cognitive Assessments (MoCA).

**Results:** Steep reductions occurred in cerebral cortical GM and deep GM nuclei volumes which correlated with each patient's clinical and cognitive impairment. The median observed percentile volume losses were statistically significant compared with the 50th percentile for each GM component. Longitudinal assessments of an unselected sample of one dozen patients involved in the PPMS study showed prominent losses occurring mainly in cortical GM and hippocampus which were reflected in their EDSS and MoCA. The longitudinal results were compared with a similar sample of patients having Relapsing MS (RMS) whose GM values were largely in normal range, annualized volume GM changes were much less, while WM hyperintensities were in abnormal range in half the unselected cases.

**Conclusions:** Knowledge of the degree and rapidity with which cortical atrophy and deep GM volume loss develops clarifies the source of progressive cognitive and clinical decline in PPMS.

## Introduction

WM abnormalities obtained on routine Brain and Spinal Cord MRI often fail to explain the source of an MS exacerbation, or the degree of cognitive and physical impairment that MS patients experience, a circumstance described as the “clinico-radiological paradox” (1, 2) Histopathologic analysis has demonstrated a substantial burden of pathology in the cerebral cortex (3–5) and deep GM structures (6) and is independent of WM demyelination (2, 6–9). Evidence has accumulated that GM and WM pathology appear to be separate, independent, and distinct (6–12). Lesions in the cerebral cortex have been visualized before WM lesions develop, and regional comparisons found cortical demyelination to be much more extensive than in WM (6, 7, 9) GM lesions, in contrast with WM, lack parenchymal lymphocyte infiltration or blood brain barrier disruption, and accumulate greater deposition of complement proteins and antibodies (13).

There is a revised understanding of the role and importance of GM changes and brain atrophy in MS largely as the result of the development of quantitative imaging techniques which provide measurements more precisely than can be done by just viewing the images. The aim of this study was to utilize NeuroQuant® 3D VBM MRI to analyze and clarify the *degree* and *longitudinal evolution* of volumetric reductions that were contributing to cognitive and clinical dysfunction in each of 25 PPMS patients. Until recently, GM lesions have been difficult to detect using conventional MRI techniques such as T2-weighted spin-echo or FLAIR images, due to the low contrast they produce as they have little T-cell related inflammation (3, 4, 14–17) or disruption of the blood brain barrier (18). Further, focal cortical lesions fail to be visualized using these conventional MR images because they tend to be small, and are difficult to distinguish from surrounding normal appearing GM (19). They also tend to have partial volume effects with WM and CSF (20). Visible cortical lesions therefore represent only “the tip of the pathologic iceberg” (21). For example, only 5% of histopathological cortical lesions were identified with standard FLAIR and T2 MRI sequences (3).

PPMS is defined among those MS patients who have insidious and inexorable progressive worsening in their clinical or cognitive function for a minimum of 12 months without recovery or remission (22, 23). The progressive worsening of disability in PPMS results from the combination of neurodegeneration and various complex immune mechanisms (23). When compared with RMS, PPMS is associated with whole brain atrophy, cortical and subpial demyelination, oxidative stress, excitotoxicity, diffuse axonal injury and microglial activation with failure to repair damaged parenchyma (24–27). Other distinguishing pathologic features which contribute to a less favorable prognosis involve exhaustion of functional compensation, lack of trophic support, and altered expression of ion channels in demyelinated axons (26). PPMS represents about 15% of the total MS patient population and, in contrast to RMS, tends to develop ~10 years later (28). Males and females are equally affected in PPMS while 70% of RMS are women (28). Histopathologic studies in PPMS have demonstrated widespread involvement of GM structures including the cerebral cortex, hippocampus, thalamus, basal ganglia, cerebellum, and spinal cord (15, 25–29).

## Methods

### Study Population

This is a retrospective longitudinal study involving a cohort of 25 PPMS patients acquired from George Washington University's Multiple Sclerosis Clinical Care database obtained between 2011 and 2020 and analyzed with 3D VBM NeuroQuant® software (NeuroQuant® v2.3, CorTechs Laboratories, San Diego, California). The patient's results were compared with a NeuroQuant® database and studied on the same MRI platform to avoid variability in findings. All the patients were restudied at ~1 year (11–14 months). They all received EDSS assessment (30) and MoCA scores (31) within 14 days of MRI acquisition. EDSS examination was performed by a single, specially trained and certified examining Neurologist. The series included 16 women, mean age of onset 30.16 ± 9.0 (13–43) years; mean disease duration 16.11 ± 11.9 (2–50) years; EDSS range between 3.0 and 6.5 at screening: mean 5.87 ± 2.59; Some patients had received other disease modifying treatments during the study period, and all patients who were participating after March, 2017 were eventually treated with ocrelizumab (32). MoCA was obtained on each subject with mean 21.32 ± 11.91 (11–28). Patients were not included who were receiving steroids or psychoactive therapy. Since all patients in the study received MR imaging as outpatients, their degree of hydration could not be standardized, and may have contributed to some of the variability in GM quantification. Similarly, as a retrospective analysis, comorbidity and daily fluctuations in brain volumes could not be factored into the results. PPMS patients were excluded who were under age 18, had EDSS <3.0, or those with other known neurological or psychiatric disorders. Screening criteria beginning at minimum of 3.0 EDSS were used based on standards established in the ocrelizumab treatment trial (32).

### MRI Acquisition

Recognizing the importance of GM pathology in PPMS, reliable methods for assessing the degree of GM pathology has become essential. 3D VBM is an advanced MRI technique which accurately quantifies brain volumes of GM structures reflecting the neurodegenerative aspects of MS, and identifies underlying structural changes not apparent with conventional MRI (14–17, 20, 33–39). These techniques have proved to be a highly reproducible means to measure brain volumes of various GM structures. There are a variety of post processing techniques using voxels available to accomplish this purpose including NeuroQuant®, which has been shown to be at statistical agreement in assessing brain volumes when compared with other validated methods such as Structural Imaging Evaluation of Normalized Atrophy (SIENAX) (40) and FreeSurfer (41). NeuroQuant® is an automated software package that provides objective quantitative analysis of regional GM volumes compared to cohort matched normative data. 3D volumetric MR imaging studies utilizes an 8 channel phased array head coil. Image acquisition includes a 3 plane localizer sequence and 3D volumetric T1 weighted gradient echo sequence. The protocol includes a 3D T1 weighted fast spoiled gradient echo sequence for volumetric measurements. Each sagittal 3D T1 volumetric image was acquired according to listed protocol https://www/cortechslabs.com/resources/technical-information/recommended-scanner-settings/ for volumetric post-processing by NeuroQuant®. Proprietary automated segmentation methods used by NeuroQuant® are evolved from widely used semi-automated methods relying on probabilistic atlas based methods to provide volumetric analysis of each segmental structure (37, 39, 40). The segmentation procedure assigns a neuroanatomic label to each voxel on the basis of probabilistic information automatically estimated from several atlases. The labeling of each point in space is achieved by finding segmentation for each voxel that maximizes the probability of input given the previous probabilities from the atlases. The software deletes non-brain tissue using active contour models and separates a number of anatomic structures using the same probabilistic atlas. The automated program compares individual volumes to a normative database taken as a percentage of total intracranial volume which is adjusted for age, and gender (37, 39, 40) allowing objective comparisons of patients with varying morphology to determine statistically significant deviations from normal for each individual. NeuroQuant® MRI automated technique quantifies whole brain, forebrain parenchyma, cortical and deep GM nuclei volumes including measures of hippocampus, thalamus, superior and inferior lateral ventricles for which age based Normative Data was available. Cerebellar volumes are available but normative data is not provided and is only included in longitudinal analysis. Quantification of WM lesion volume and Normative Data are obtained directly from the NeuroQuant® dataset and identified as WM hypointensities.

**Figure 1:**
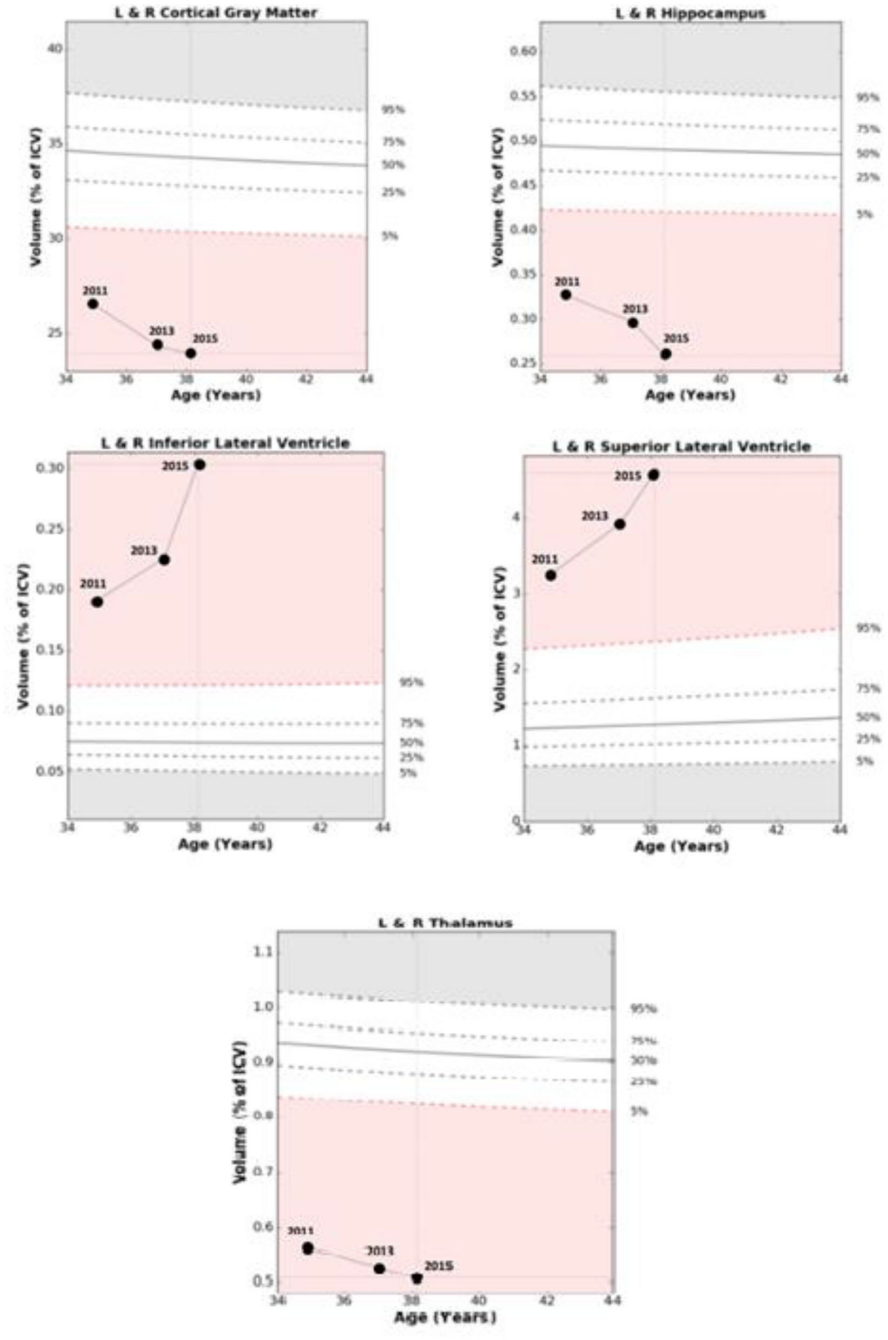
Comparisons of age related Normative Data based on percentage of intracranial volume with relative changes over 39 months in Case 1. Abnormal range is defined as out of the white zone at the 5th percentile or less, or 95th percentile or more based at a given age.

**Figure 2:**
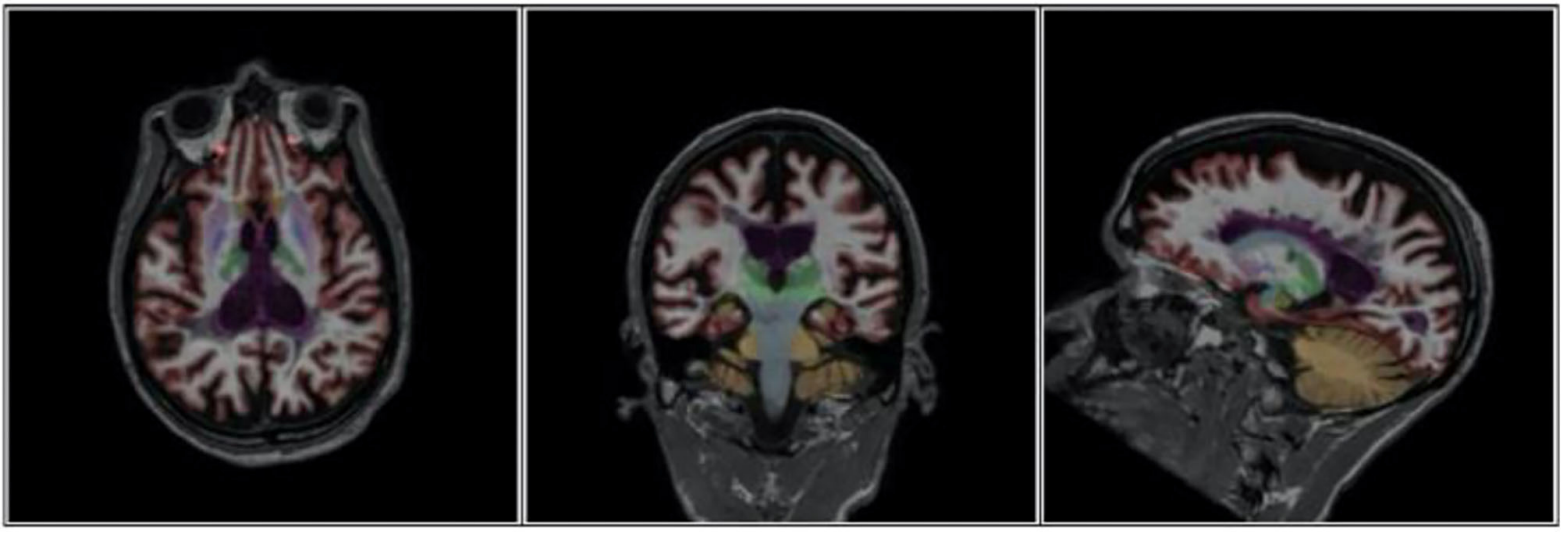
Axial, Coronal and Sagittal images of Case 1 demonstrating extent of brain atrophy as documented in 2015.

An example of NeuroQuant® numeric data acquisition and morphometry results which track GM volumes and ventricular size with progression over time is shown in Table 1 and Figure 1. Representative axial, coronal, and sagittal MRI images of case 1 from 2015 are shown in Figure 2.

**Table 1:** NeuroQuant® Volumetric and Normative Data from a patient acquired between October, 2011 and January, 2015 showing degree and progression of volume loss in GM brain structures and increase in ventricular size over time.

**Rate of Progression 2011–2015**					
	**Oct. 2011 volume (cm^3^)**	**Dec. 2013 volume (cm^3^)**	**Jan. 2015 volume (cm^3^)**	**2011-15 39 month percent change**	**(2015) normative percentile for patient's age**
Whole brain	1,035.08	1,056.61	1,008.72	−2.25%	1
Forebrain parenchyma	877.37	907.69	853.64	−2.70%	1
Cortical gray matter	410.38	379.1	367.81	−10.37%	1
Superior lateral ventricles	50.33	61.2	70.72	+40.51%	99
Hippocampus	4.95	4.63	3.93	−20.61%	1
Inferior lateral ventricles	2.9	3.51	4.37	+50.69%	99
Thalamus	8.83	8.35	7.55	−14.50%	1

### Statistical Analysis (Table 2)

Non-parametric 1-sample sign test was used to examine the difference between the median of the observed percentile scores and the expected score at the 50th percentile. The findings were that the median observed percentiles differed in a statistically significant value from the 50th percentile for each GM structure, as well as the size of the ventricles, where normative data was available

**Table 2:** Sign test NORMATIVE results by brain region.

**Region**	**Mean (SD) percentile**	**Sign test *p* for difference from 50th percentile**
Whole brain	10.1 ± 21.1	<0.0001
Cortical gray	1.3 ± 0.8	<0.0001
Forebrain parenchyma	8.0 ± 18.6	<0.0001
Thalamus	8.9 ± 21.3	0.0007
Hippocampus	19.4 ± 25.2	0.0044
Superior lateral ventricle	97.4 ± 2.4	<0.0001
Inferior lateral ventricle	96.6 ± 5.1	<0.0001

## Results (Table 3)

### Cross Sectional Analysis

The most notable results in this study were the abnormal GM Normative Data present in all 25 PPMS patients. Among these study patients, those that were found to have GM structure volumes out of the normal range at or less than the 5th percentile for their age were: whole brain 14; cortical GM 23; forebrain parenchyma 17; and thalamus 21. There were 10 patients with hippocampal volumes at or less than the 5th percentile, but another 5 were minimally above the abnormal range at 6, 9, and three at 11th percentile. Cerebellar results are not reported as Normative Data is not available in the NeuroQuant® database but longitudinal change in values is presented in 12 randomly selected patients in Supplement A. The superior and inferior lateral ventricles were both found to be out of the normal range at or greater than the 95th percentile in 20 patients. There was a rough correlation between EDSS scores and the volume of white matter hyperintensities with 18 patients with values at or exceeding the 95th percentile.

**Table 3:** Summary of 25 PPMS patient's clinical characteristics and Normative Range results using their most recent NeuroQuant® database.

**Patient characteristics with NeuroQuant® data**												
	**Age of onset (year) and sex**	**MoCA**	**Duration of disease (years)**	**EDSS**	**Whole brain**	**Cortical gray**	**Fore brain Parenchyma**	**Thalamus**	**Hippo campus**	**Superior lateral ventricles**	**Inferior lateral ventricles**	**WM hypo-intensities**
1	36 M	11	5	8	1	1	1	1	1	99	99	99
2	22 F	19	2	7	1	1	1	1	2	99	90	99
3	19 F	19	50	6.5	1	1	1	80	1	99	99	99
4	34 M	24	6	6	39	1	32	56	38	95	79	99
5	23 F	28	13	8	1	1	1	1	60	97	96	99
6	23 F	23	7	4	4	1	2	5	29	99	99	96
7	33 F	21	9	4	7	1	3	1	18	99	99	99
8	40 F	21	15	5.5	1	1	1	1	1	92	99	98
9	42 F	19	5	4	1	1	1	1	6	99	99	83
10	13 M	13	17	8.5	89	1	79	1	19	97	91	99
11	25 F	23	38	4	17	4	10	6	53	93	99	87
12	19 F	22	16	4	1	2	1	1	89	99	99	99
13	38 M	19	19	6.5	5	1	5	1	1	99	95	99
14	32 M	22	23	7	3	3	2	1	11	93	97	99
15	37 F	21	14	7.5	1	1	1	1	2	97	99	99
16	43 F	20	21	4.5	8	1	4	1	2	99	99	91
17	23 F	25	9	7	1	1	1	1	5	98	99	99
18	31 F	28	12	4.5	3	1	2	9	36	99	99	91
19	40 F	27	25	5	8	1	4	1	2	99	99	96
20	28 M	29	9	3	1	1	3	1	18	91	92	79
21	40 M	18	25	5	9	1	9	1	1	99	93	91
22	30 F	23	17	5.5	24	2	15	1	11	99	99	93
23	13 M	19	19	8	89	1	71	1	99	97	96	99
24	33 M	23	24	6.5	14	7	17	1	9	93	97	99
25	50 F	23	9	6	8	8	24	1	11	99	99	99

### Longitudinal Analysis (Supplements A,B)

Over 1 year of longitudinal data collection, patients with PPMS showed a % decrease in volume in a number of GM structures, but losses were most prominent in cortical GM (−7.7 SD 10.2) and hippocampus (−6.6 SD 8.2).

Supplement A provides details of a longitudinal analysis for a random group of 12 PPMS study patients, and in Supplement B, a similar number of unselected RMS cases collected over a number of years for comparison. The results provide a striking contrast as RMS patients have GM values mostly in normal range when compared to PPMS and relatively small annualized volume changes. The major variant from normal among these RMS patients is that half were found to have white matter hypointensities at or in excess of the 95th percentile which generally tended to correlate with their EDSS.

## Discussion

WM pathology has been the major focus of MS clinical assessment and research because of the ease of detection on standard MRI despite aspects such as physical disability, cognitive impairment, fatigue and seizures which link to GM dysfunction (42). Conventional neuroimaging techniques may not provide adequate explanations for the existing symptomatology, or the clinical evolution of the disease (1–3). Amiri et al. emphasized that a keener understanding of the neuropathological correlates resulting from whole brain and deep GM atrophy is urgently required (38). They concluded that advanced 3D VBM neuroimaging techniques have allowed for the assessment of GM pathology *in vivo* which correlates closely with clinical disability and cognitive impairment and overcomes many of the limitations of conventional neuroimaging (38).

Neocortical neuronal loss has been shown to be “massive” in *post mortem* examination of the brains of PPMS patients and strongly correlates with reductions of cortical volume (9, 13). Histopathologic study identified neuro-axonal loss and neuronal shrinkage as the source for regional cortical atrophy (9). Cortical atrophy appeared to be unrelated to the degree of myelin loss although such loss can be extensive in PPMS (3, 9).

GM atrophy, involving both cortical and deep GM structures, progresses more rapidly than WM atrophy, and is mainly responsible for losses in whole brain volume (33, 43, 44). Atrophy of GM structures were detected at the earliest stages of PPMS (33, 45). Whole brain atrophy, occurs at a faster pace, is more severe, extensive, and a stronger predictor of disability than WM atrophy (45–48). It is unknown is whether GM volume loss occurs independently, or results from WM axonal transection with retrograde degeneration (49). Both cortical lesions and GM atrophy are independent predictors of cognitive impairment (50–54), epilepsy (55), fatigue (56, 57), depression (58), and disability (59–62). Assessment of baseline cognitive function was of less value in predicting the probability of future cognitive decline as were MRI measures (61–63). In a serial study involving 1214 MS patients afflicted with all phenotypes and with average follow up of 2.42 years, deep GM volume loss was their main source of disability (45). The decline in deep GM volumes among their 125 patients with PPMS was found to be 1.66% annually (45).

The magnitude of whole brain atrophy in PPMS was found to be more pronounced than in RMS which was more reflective of GM atrophy rather than WM loss (29, 43, 64). Whole brain atrophy results from permanent and irreversible loss of brain parenchyma, which increases with the decline in clinical and cognitive functions (43).

Eijlers et al. demonstrated that cortical atrophy proceeded 1.8 times faster in progressive forms of MS as compared to RMS with higher regional atrophy rates for the parietal, occipital and frontal lobes (54). Cortical GM is an independent predictor of cognitive impairment and disability (52, 54, 65, 66). The most remarkable aspect of the present study is the vulnerability of cortical GM in all but two of our 25 PPMS patients with each having documented volumes below the normative range at or less than 5th percentile.

The hippocampus plays a crucial role in episodic memory formation and retrieval, and hippocampal cell loss can account for the cognitive decline present in over half of MS patients (67).

**Figure 3:**
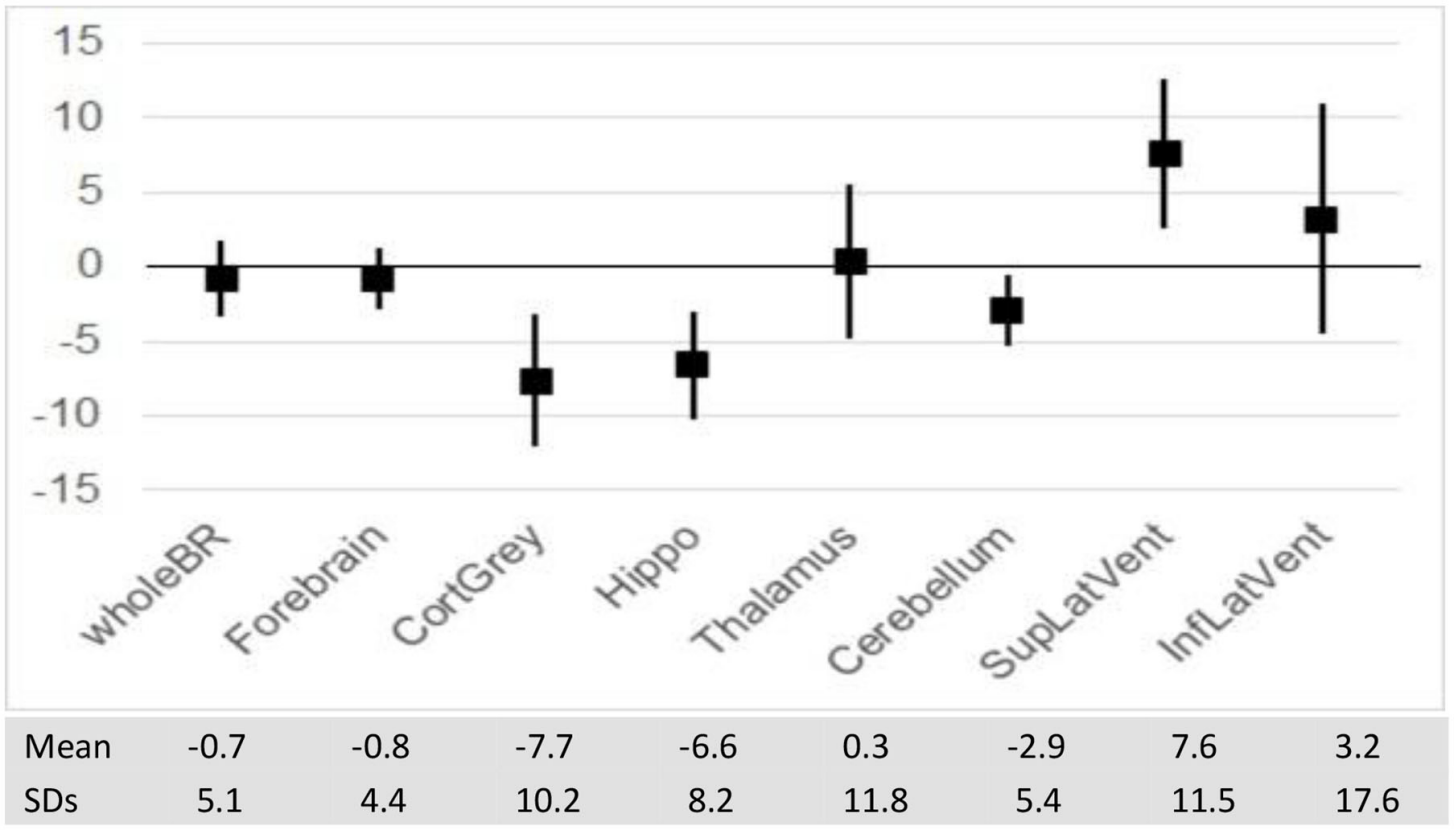
Mean per-cent change in Brain volumes and ventricular size over 1 year (with SD).

The extent of lesions involving the hippocampus has correlated with impaired visuospatial memory performance (68, 69). Recent studies have reported substantial atrophy with neuronal loss in the hippocampus of all phenotypes of MS patients (70–72). In a study among 45 patients with PPMS, a majority of hippocampal lesions had synaptic loss that was more extensive than neuronal reduction and independent of focal demyelination (73). Hippocampal volumes were smaller in MS patients when compared to healthy controls, and consistent with the degree of depression that MS patients were experiencing (70). One of our study patients experienced a remarkable 20.61% decline in hippocampal volume over 39 months (Table 1). Overall, the ~1 year longitudinal studies of our 25 PPMS patients showed an average 6.6 % loss in hippocampal volume (Figure 3).

The thalamus is involved in all major functional circuits in the brain and mediates a variety of cognitive activities (74–80). Therefore, thalamic volume loss is one of the most clinically relevant consequences of the disease (67, 72) and dysfunction or atrophy of thalamus can result in cognitive dysfunction and fatigue (74–80). Thalamic involvement is associated with a wide variety of other clinical manifestations including motor deficits, chronic pain syndromes, and abnormalities in control of eye movements (74–80). Thalamic atrophy can be one of the earliest and most evident signs of MS pathology (79, 81) and the rate of atrophy remains high throughout the course of the disease in PPMS (81). Some studies demonstrated worsening disability proportional to the extent of thalamic damage (74–80). 3D VBM MRI scanning was used to assess GM structures including thalamic volumes in 79 MS patients and compared with 16 healthy controls (77). There was a 16.8% reduction in thalamic volume in MS patients which correlated with their impaired cognitive performance. 3D VBM analysis was used to examine regional distribution of GM atrophy in 31 patients with PPMS and identified increased thalamic atrophy compared with healthy controls (74). Pontillo et al. showed that PPMS patients had more significant reductions in thalamus when compared to RMS largely due to local microstructural damage and was the most accurate predictor of cognitive impairment (81). Thalamic and cortical gray atrophy have been implicated in objective cognitive impairment as determined by NeuroQuant® analysis in a study by Kletenik et al. (75) They found that among 158 patients who completed Quality of Life in Neurologic Disorders Measures (Neuro-QoL), those with cognitive concerns were associated with reduced thalamic and cortical GM volumes. No other analyzed areas of interest correlated with cognitive issues.

## Conclusions

The importance of GM integrity in preserving clinical and cognitive function makes it essential to have an accurate means of measuring the degree of GM pathology. New techniques employing 3D VBM MRI have improved specificity and sensitivity in monitoring the anatomic substrate for a patient's clinical condition (20, 37, 38, 40, 41, 72, 82–84). These quantitative MR applications have allowed precise measurements of GM tissue damage that lead to disability accumulation. Automated 3D VBM techniques have been shown to perform as well as, or better than, manual segmentation performed by expert Neuroradiologists, Radiologists and Neurologists, who have had specialized training and expertise in anatomic labeling of MR images (40, 41, 82, 84). NeuroQuant® was able to provide a precise and reproducible means of measuring volumes of cortical GM and deep GM nuclei, with an easily interpretable imaging technique (37, 40, 41). NeuroQuant® analysis documented widespread atrophy occurring in whole brain, cortical GM, forebrain parenchyma, hippocampus, and thalamus for which Normative Data was available, and which correlated with each patient's clinical and cognitive status. Upon analyzing the Normative Data from 25 PPMS patients, most had statistically significant abnormal GM volumes measuring at or less than the 5th percentile, as well as enlargement of superior and inferior ventricular volumes at or greater than the 95th percentile for their age (Table 2). These results suggest that 3D VBM thereby reduced some of the uncertainty associated with the “clinico-radiological paradox” (1, 2).

Over a period of ~1 year (11–14 months) longitudinal analysis disclosed reductions in cortical GM and hippocampus volumes when compared to baseline values. A longitudinal sample of unselected RMS patients were compared, and differed considerably as their GM volumes were largely preserved, had much lower annualized changes, and their only variance was that the levels of white matter hypointensities were out of normal range in half the patients (Supplement B). These results warrant further research and verification by studying cerebral cortical GM and deep GM nuclei atrophy in larger cohorts of PPMS and RMS patients.

Serial 3D VBM study could be utilized to assess efficacy in experimental MS disease modifying drug trials. In a clinical setting, longitudinal GM volume analysis would help to establish whether patients meet the criteria for “No Evidence of Disease Activity (NEDA)” which has emerged as the therapeutic target for disease modifying therapies (85).

This study has some limitations. Serious shortcomings are identified in the segmentation of deep GM structures using automated techniques (44, 48, 71, 86). There are biologic confounders which could influence the analysis of GM volumes including the introduction of disease modifying therapies, physiologic factors such as state of hydration, normal aging, comorbidities, and daily fluctuations in brain volumes (12, 86). The sample size is small and requires confirmation by encompassing larger numbers of PPMS patients utilizing NeuroQuant® or other 3D VBM automated techniques. Nevertheless, the results of this study are of interest because they underscore the statistically significant GM volume losses outside the normal range for each individual PPMS patient.

In conclusion, the use of 3D VBM MRI analysis and longitudinal study has contributed to a fuller understanding of PPMS pathophysiology *in vivo* and the prominent role of GM involvement. Knowledge of the degree and rapidity with which cortical atrophy and deep GM volume loss develops clarifies the source of progressive cognitive and clinical decline in PPMS.

## Data Availability Statement

The raw data supporting the conclusions of this article will be made available by the authors, without undue reservation.

## Ethics Statement

The studies involving human participants were reviewed and approved by George Washington University Institutional Review Board. Written informed consent for participation was not required for this study in accordance with the national legislation and the institutional requirements.

## Author Contributions

TR is responsible for all aspects of this manuscript.

## Conflict of Interest

The author declares that the research was conducted in the absence of any commercial or financial relationships that could be construed as a potential conflict of interest.
