# Supplementary material for: Gray Matter Matters: A Longitudinal Magnetic Resonance Voxel-Based Morphometry Study of Primary Progressive Multiple Sclerosis
Source: Front Neurol. 2020 Nov 12;11:581537. doi: 10.3389/fneur.2020.581537 (PMC7689315; doi:10.3389/fneur.2020.581537)
Supplement: Supplementary file 1 [file Data_Sheet_1.docx]

**SUPPLEMENT A. NeuroQuant data on random PPMS patients**

**TABLE 1a. Patient 1.**

|  | Dec 2013  Volume  (cm³) | Jan. 2015  Volume  (cm³) | 13 month  Percent  Change | 2015  Normative  Percentile For  Patient’s Age |
| --- | --- | --- | --- | --- |
| Whole Brain | 1056.61 | 1008.72 | -4.53% | 1 |
| Forebrain Parenchyma | 907.69 | 853.64 | -5.95% | 1 |
| Cortical Grey Matter | 379.1 | 367.81 | -2.98% | 1 |
| Superior Lateral Ventricles | 61.2 | 70.72 | +15.6% | 99 |
| Hippocampus | 4.63 | 3.93 | -15.1% | 1 |
| Inferior Lateral Ventricles | 3.51 | 4.37 | +24.5% | 99 |
| Thalamus | 8.35 | 7.55 | -9.58% | 1 |
| Cerebellum | 138.19 | 135.48 | -1.53% | NA |
| White Matter Hypointensity | 29.23 | 32.6 | +11.5% | 99 |
| MoCA | 13 | 11 |  |  |
| EDSS | 8.0 | 8.0 |  |  |

**TABLE 2a. Patient 2.**

|  | July 2018  Volume  (cm³) | Aug 2019  Volume  (cm³) | 13 month  Percent  Change | 2019 Normative  Percentile For  Patient’s Age |
| --- | --- | --- | --- | --- |
| Whole Brain | 990.36 | 963.95 | -2.67% | 1 |
| Forebrain Parenchyma | 859.6 | 831.79 | -3.09 | 1 |
| Cortical Grey Matter | 308.14 | 280.97 | -8.8% | 1 |
| Superior Lateral Ventricles | 44.22 | 42.00 | -5.04% | 99 |
| Hippocampus | 5.91 | 6.00 | +1.5% | 1 |
| Inferior Lateral Ventricles | 1.34 | 1.46 | +8.96% | 99 |
| Thalamus | 7.18 | 6.87 | -4.3% | 1 |
| Cerebellum | 113.51 | 110.56 | -2.65% | NA |
| White Matter Hypointensity | 18.3 | 28.0 | +53.0% | 99 |
| MoCA | 21 | 19 |  |  |
| EDSS | 7.5 | 8.0 |  |  |

**TABLE 3a. Patient 15.**

|  | July 2018  Volume  (cm³) | June 2019  Volume  (cm³) | 11 month  Percent  Change | 2019  Normative  Percentile For  Patient’s Age |
| --- | --- | --- | --- | --- |
| Whole Brain | 849.17 | 868.06 | +2.35% | 1 |
| Forebrain Parenchyma | 706.85 | 719.0 | +1.72% | 1 |
| Cortical Grey Matter | 286.34 | 246.38 | -14.25% | 1 |
| Superior Lateral Ventricles | 25.41 | 28.68 | +12.85% | 99 |
| Hippocampus | 6.25 | 5.06 | -19.05% | 2 |
| Inferior Lateral Ventricles | 1.58 | 1.36 | -13.99% | 99 |
| Thalamus | 7.26 | 6.63 | -8.72% | 1 |
| Cerebellum | 129.3 | 123.8 | -4.33% | NA |
| White Matter Hypointensity | 18.3 | 28.0 | +53% | 99 |
| MoCA | 21 | 21 |  |  |
| EDSS | 7.5 | 7.5 |  |  |

**TABLE 4a. Patient 21.**

|  | Dec 2018  Volume  (cm³) | Nov 2019  Volume  (cm³) | 11 month  Percent  Change | 2019  Normative  Percentile For  Patient’s Age |
| --- | --- | --- | --- | --- |
| Whole Brain | 1081.5 | 1088.5 | +0.65% | 9 |
| Forebrain Parenchyma | 940.23 | 944.7 | +0.48% | 9 |
| Cortical Grey Matter | 381.3 | 379.4 | -0.47% | 1 |
| Superior Lateral Ventricles | 60.6 | 58.9 | -2.80% | 99 |
| Hippocampus | 5.6 | 5.36 | -4.3% | 1 |
| Inferior Lateral Ventricles | 2.33 | 2.21 | -5.15% | 93 |
| Thalamus | 10.2 | 10.4 | +1.96% | 1 |
| Cerebellum | 122.3 | 124.75 | +2.0% | NA |
| White Matter Hypointensity | 13.8 | 18.2 | +31.9% | 99 |
| MoCA | 20 | 18 |  |  |
| EDSS | 5.0 | 5.0 |  |  |

**TABLE 5a. Patient 6**

|  | June  2018  Volume  (cm³) | Aug. 2019  Volume  (cm³) | 14 month  Percent  Change | 2019  Normative  Percentile For  Patient’s Age |
| --- | --- | --- | --- | --- |
| Whole Brain | 943.4 | 930.9 | -1.32% | 1 |
| Forebrain Parenchyma | 810.18 | 792.8 | -2.15% | 2 |
| Cortical Grey Matter | 334.1 | 323.2 | -3.26% | 1 |
| Superior Lateral Ventricles | 53.4 | 54.19 | +1.48% | 99 |
| Hippocampus | 6.28 | 6.32 | +0.64% | 29 |
| Inferior Lateral Ventricles | 1.75 | 1.84 | +5.14% | 99 |
| Thalamus | 10.2 | 10.38 | +0.1% | 1 |
| Cerebellum | 115.8 | 115.8 | 0% | NA |
| White Matter Hypointensity | 19.4 | 20.9 | +7.73% | 99 |
| MoCA | 23 | 23 |  |  |
| EDSS | 6.0 | 6.0 |  |  |

**TABLE 6a. Patient 14**

|  | Oct 2018  Volume  (cm³) | Nov 2019  Volume  (cm³) | 13 month  Percent  Change | 2019  Normative  Percentile For  Patient’s Age |
| --- | --- | --- | --- | --- |
| Whole Brain | 1137.22 | 1110.39 | -2.36% | 3 |
| Forebrain Parenchyma | 991.37 | 957.98 | -3.37 % | 2 |
| Cortical Grey Matter | 465.8 | 450.9 | -3.2% | 3 |
| Superior Lateral Ventricles | 44.9 | 44.9 | 0% | 93 |
| Hippocampus | 6.48 | 6.57 | +1.4% | 11 |
| Inferior Lateral Ventricles | 2.50 | 2.54 | +1.6% | 97 |
| Thalamus | 10.8 | 10.35 | -4.2% | 1 |
| Cerebellum | 134.08 | 128.62 | -4.1% | NA |
| White Matter Hypointensity | 7.96 | 6.98 | -12.3% | 99 |
| MoCA | 22 | 22 |  |  |
| EDSS | 7.0 | 7.0 |  |  |

**TABLE 7a. Patient 9.**

|  | Sept 2017  Volume  (cm³) | Sept 2018  Volume  (cm³) | 12 month  Percent  Change | 2018  Normative  Percentile For  Patient’s Age |
| --- | --- | --- | --- | --- |
| Whole Brain | 1369.74 | 1269.8 | -7.3% | 1 |
| Forebrain Parenchyma | 1193.1 | 1102.5 | -7.6 % | 1 |
| Cortical Grey Matter | 491.1 | 426.1 | -13.3% | 1 |
| Superior Lateral Ventricles | 92.6 | 102.5 | +10.7% | 99 |
| Hippocampus | 6.27 | 7.62 | +21.5% | 6 |
| Inferior Lateral Ventricles | 3.34 | 2.57 | -23.1% | 99 |
| Thalamus | 9.98 | 11.2 | +12.2% | 1 |
| Cerebellum | 155.7 | 147.2 | -5.6% | NA |
| White Matter Hypointensity | 35.83 | 66.07 | +84.4% | 99 |
| MoCA | 19 | 19 |  |  |
| EDSS | 3.5 | 4.0 |  |  |
|  |  |  |  |  |

**TABLE 8a. Patient 7.**

|  | Oct 2018  Volume  (cm³) | Nov 2019  Volume  (cm³) | 13 month  Percent  Change | 2019  Normative  Percentile For  Patient’s Age |
| --- | --- | --- | --- | --- |
| Whole Brain | 946.3 | 937.7 | -0.91% | 7 |
| Forebrain Parenchyma | 808.7 | 798.6 | -1.25 % | 3 |
| Cortical Grey Matter | 375.9 | 360.1 | -4.2% | 1 |
| Superior Lateral Ventricles | 40.59 | 43.6 | +7.4% | 99 |
| Hippocampus | 5.89 | 6.01 | +2.0% | 6 |
| Inferior Lateral Ventricles | 2.08 | 2.36 | +13.5% | 99 |
| Thalamus | 8.72 | 8.36 | -4.12% | 1 |
| Cerebellum | 119.99 | 110.8 | -0.58% | NA |
| White Matter Hypointensity | 6.0 | 6.38 | +7.67% | 99 |
| MoCA | 22 | 21 |  |  |
| EDSS | 4.0 | 4.0 |  |  |

**TABLE 9a. Patient 12**

|  | Oct 2017  Volume  (cm³) | Nov 2018  Volume  (cm³) | 13 month  Percent  Change | 2018  Normative  Percentile For  Patient’s Age |
| --- | --- | --- | --- | --- |
| Whole Brain | 966.47 | 987.8 | +2.2% | 1 |
| Forebrain Parenchyma | 830.56 | 835.18 | +0.56% | 1 |
| Cortical Grey Matter | 407.96 | 422.4 | +3.5% | 2 |
| Superior Lateral Ventricles | 34.85 | 35.3 | +1.29% | 99 |
| Hippocampus | 7.82 | 7.88 | +0.77% | 89 |
| Inferior Lateral Ventricles | 1.82 | 2.13 | +17.03% | 99 |
| Thalamus | 9.22 | 10.0 | +8.5% | 1 |
| Cerebellum | 106.87 | 115.91 | +8.46% | NA |
| White Matter Hypointensity | 26.25 | 29.47 | +12.27% | 99 |
| MoCA | 21 | 22 |  |  |
| EDSS | 3.5 | 4.0 |  |  |

**TABLE 10a. Patient 17.**

|  | Oct 2018  Volume  (cm³) | Nov 2019  Volume  (cm³) | 13 month  Percent  Change | 2019  Normative  Percentile For  Patient’s Age |
| --- | --- | --- | --- | --- |
| Whole Brain | NA | 942.98 | NA | 1 |
| Forebrain Parenchyma | 933.9 | 843.13 | -9.7% | 1 |
| Cortical Grey Matter | 509.69 | 382.72 | -24.9% | 1 |
| Superior Lateral Ventricles | 39.07 | 72.72 | +86.1% | 98 |
| Hippocampus | 7.81 | 6.39 | -18.3% | 5 |
| Inferior Lateral Ventricles | 2.20 | 2.25 | +12.5% | 99 |
| Thalamus | 14.71 | 10.06 | -31.65% | 1 |
| Cerebellum | 102.95 | 81.29 | -21.3% | NA |
| White Matter Hypointensity | NA | 19.33 | NA | 99 |
| MoCA | 26 | 25 |  |  |
| EDSS | 5.5 | 7.0 |  |  |

**TABLE 11a. Patient 25.**

|  | Oct 2018  Volume  (cm³) | Nov 2019  Volume  (cm³) | 13 month  Percent  Change | 2019  Normative  Percentile For  Patient’s Age |
| --- | --- | --- | --- | --- |
| Whole Brain | 1021.46 | 1089.85 | +6.7% | 8 |
| Forebrain Parenchyma | 963.77 | 934.72 | -4.0% | 24 |
| Cortical Grey Matter | 450.2 | 451.4 | +0.49% | 8 |
| Superior Lateral Ventricles | 89.5 | 107.4 | +20.0% | 99 |
| Hippocampus | 6.93 | 6.52 | -5.9% | 11 |
| Inferior Lateral Ventricles | 3.01 | 4.12 | +36.87% | 99 |
| Thalamus | 10.39 | 11.06 | +6.45% | 1 |
| Cerebellum | 137.49 | 134.57 | -2.13% | NA |
| White Matter Hypointensity | 9.31 | 9.61 | +3.3% | 99 |
| MoCA | 22 | 22 |  |  |
| EDSS | 6.0 | 6.5 |  |  |

**Table 12a. Patient 18.**

|  | June 2018  Volume  (cm³) | Aug 2019  Volume  (cm³) | 14 month  Percent  Change | 2019  Normative  Percentile For  Patient’s Age |
| --- | --- | --- | --- | --- |
| Whole Brain | 943.36 | 930.89 | -1.43% | 3 |
| Forebrain Parenchyma | 810.18 | 797.8 | -1.53% | 2 |
| Cortical Grey Matter | 334.1 | 323.2 | -3.26% | 1 |
| Superior Lateral Ventricles | 53.36 | 54.19 | +1.56% | 99 |
| Hippocampus | 6.28 | 6.29 | +0.16% | 36 |
| Inferior Lateral Ventricles | 1.75 | 1.84 | +5.14% | 99 |
| Thalamus | 10.20 | 10.29 | +2.9% | 9 |
| Cerebellum | 115.8 | 113.8 | -1.73% | NA |
| White Matter Hypointensity | 19.43 | 20.89 | +7.51% | 99 |
| MoCA | 28 | 28 |  |  |
| EDSS | 4.0 | 4.5 |  |  |
