# Supplementary material for: Gray Matter Matters: A Longitudinal Magnetic Resonance Voxel-Based Morphometry Study of Primary Progressive Multiple Sclerosis
Source: Front Neurol. 2020 Nov 12;11:581537. doi: 10.3389/fneur.2020.581537 (PMC7689315; doi:10.3389/fneur.2020.581537)
Supplement: Supplementary file 2 [file Data_Sheet_2.docx]

**SUPPLEMENT B. LONGITUDINAL DATA ON PATIENTS WITH RELAPSING REMITTING MULTIPLE SCLEROSIS**

**Table 1b. Patient 1**

|  | April 2018  Volume  (cm³) | Aug 2019  Volume  (cm³) | 16 month  Percent  Change | Annualized  Percentage  change | Normative Percentile  For  Patient’s  Age |
| --- | --- | --- | --- | --- | --- |
| Cortical Grey Matter | 446.94 | 437.27 | -2.16% | -1.62 | 28 |
| Superior Lateral Ventricles | 42.19 | 42.93 | +1.75% | +1.31 | 88 |
| Hippocampus | 7.28 | 7.14 | -1.92% | -1.44 | 81 |
| Inferior Lateral Ventricles | 2.23 | 2.28 | +2.24% | -1.68 | 85 |
| Thalamus | 14.13 | 14.25 | +0.85% | +0.64 | 97 |
| Cerebellum | NA | 111.05 |  |  | NA |
| White Matter Hypointensity | 3.84 | 3.29 | -14.84% | +11.3 | 97 |
| EDSS | 4.0 | 4.0 |  |  |  |

**Table 2b. Patient 2.**

|  | July 2015 Volume  (cm³) | May 2018  Volume  (cm³) | 34 month  Percent  Change | Annualized  Percentage  change | 2018 Normative Percentile For Patient’s Age |
| --- | --- | --- | --- | --- | --- |
| Cortical Grey Matter | 494.66 | 507.0 | +2.49% | +0.88 | 75 |
| Superior Lateral Ventricles | 23.21 | 20.24 | -12.8% | -4.51 | 31 |
| Hippocampus | 7.78 | 8.07 | +3.73% | +1.31 | 72 |
| Inferior Lateral Ventricles | 1.03 | 0.7 | -32.0% | -11.29 | 16 |
| Thalamus | 14.0 | 13.68 | -2.29% | -0.80 | 62 |
| Cerebellum | 145.68 | 139.19 | -4.45% | -1.57 | NA |
| White Matter Hypointensity | NA | 0.73 | NA | NA | 85 |
| EDSS | 5.0 | 6.0 |  |  |  |

**Table 3b. Patient 3.**

|  | April 2019  Volume  (cm³) | Mar 2020  Volume  (cm³) | 11 month  Percent  Change | Annualized  Percentage  change | 2020  Normative  Percentile  For  Patient’s  Age |
| --- | --- | --- | --- | --- | --- |
| Cortical Grey Matter | 428.4 | 440.8 | +2.89% | +3.15 | 21 |
| Superior Lateral Ventricles | 28.67 | 27.9 | -2.69% | -2.93 | 88 |
| Hippocampus | 6.72 | 6.64 | -1.2% | -1.31 | 37 |
| Inferior Lateral Ventricles | 1.48 | 1.33 | -10.1% | -11.02 | 86 |
| Thalamus | 11.74 | 12.53 | +6.72% | +7.33 | 83 |
| Cerebellum | 103.6 | 114.21 | +10.24% | +11.17 | NA |
| White Matter Hypointensity | 1.34 | 1.06 | -20.82% | -22.71 | 86 |
| EDSS | 2.5 | 2.5 |  |  |  |

**Table 4b. Patient 4.**

|  | July 2019  Volume  (cm³) | June 2020  Volume  (cm³) | 11 month  Percent  Change | Annualized  Percentage  change | 2020  Normative Percentile  For  Patient’s  Age |
| --- | --- | --- | --- | --- | --- |
| Cortical Grey Matter | 458.36 | 447.1 | -2.46% | -2.68 | 26 |
| Superior Lateral Ventricles | 14.55 | 13.74 | -5.57% | -6.08 | 17 |
| Hippocampus | 7.47 | 7.10 | -4.9% | -5,35 | 69 |
| Inferior Lateral Ventricles | 0.84 | 0.94 | +11.9% | +12.98 | 32 |
| Thalamus | 14.69 | 14.35 | -2.31% | -2.52 | 99 |
| Cerebellum | 119.6 | 107.9 | -9.78 | -10.67 | NA |
| White Matter Hypointensity | 0.3 | 0.31 | +3.33 | +3.63 | 35 |
| EDSS | 3.5 | 4.0 |  |  |  |

**Table 5b. Patient 5.**

|  | July 2014  Volume  (cm³) | July 2019  Volume  (cm³) | 60 month  Percent  Change | Annualized  Percentage  change | 2019  Normative Percentile For  Patient’s  Age |
| --- | --- | --- | --- | --- | --- |
| Cortical Grey Matter | 434.2 | 428.5 | -1.96 | -0.39 | 4 |
| Superior Lateral Ventricles | 43.77 | 49.91 | +14.02 | +2.80 | 99 |
| Hippocampus | 7.42 | 6.97 | -9.13 | -1.83 | 44 |
| Inferior Lateral Ventricles | 2.75 | 3.07 | +11.6 | +2.32 | 99 |
| Thalamus | 10.4 | 11.9 | +6.54 | +1.3 | 32 |
| Cerebellum | 129.1 | 127.9 | -0.93 | -0.19 | NA |
| White Matter Hypointensity | 10.6 | 12.3 | +16.03 | +3.38 | 99 |
| EDSS | 2.5 | 4.0 |  |  |  |

**Table 6b. Patient 6.**

|  | Feb 2014  Volume  (cm³) | April 2017  Volume  (cm³) | 38 month  Percent  Change | Annualized  Percentage  change | 2017  Normative  Percentile  For Patient’s  Age |
| --- | --- | --- | --- | --- | --- |
| Cortical Grey Matter | 406.3 | 374.4 | -7.85% | -2.48 | 1 |
| Superior Lateral Ventricles | 11.8 | 15.53 | +31.61% | +9.98 | 35 |
| Hippocampus | 7.64 | 6.47 | - 15.3% | -4.83 | 48 |
| Inferior Lateral Ventricles | 1.06 | 2.69 | +153.77% | +48.54 | 66 |
| Thalamus | 12.2 | 11.3 | -7.38% | -2.33 | 6 |
| Cerebellum | 136.0 | 116.94 | -14.01% | -4.42 | NA |
| White Matter Hypointensity | NA | 1.59 | NA | NA | 96 |
| EDSS | 2.0 | 2.5 |  |  |  |

**Table 7b. Patient 7**

|  | Oct 2014  Volume  (cm³) | Nov 2018 Volume  (cm³) | 49 month  Percent  Change | Annualized  Percentage  Change | 2018  Normative Percentage  For Patient’s  Age |
| --- | --- | --- | --- | --- | --- |
| Cortical Grey Matter | 549.1 | 510.24 | -7.08% | -1.73 | 21 |
| Superior Lateral Ventricles | 32.75 | 30.27 | -7.57% | -1.85 | 94 |
| Hippocampus | 7.43 | 6.96 | -6.32% | -1.55 | 14 |
| Inferior Lateral Ventricles | 2.13 | 1.74 | -18.3% | -4.48 | 94 |
| Thalamus | 11.75 | 12.5 | +6.38 | +1.56 | 1 |
| Cerebellum | 121.4 | 121.4 | 0 |  | NA |
| White Matter Hypointensity | 8.23 | 7.35 | -10.69% | -2.62 | 99 |
| EDSS | 2.0 | 1.5 |  |  |  |

**Table 8b. Patient 8.**

|  | Oct 2016  Volume  (cm³) | Sept 2018  Volume  (cm³) | 23 month  Percent  Change | Annualized  Percentage  change | Normative Percentile For  Patient’s Age |
| --- | --- | --- | --- | --- | --- |
| Cortical Grey Matter | 419.22 | 418.10 | -0.27% | -0.14 | 18 |
| Superior Lateral Ventricles | 17.4 | 13.6 | -21.9% | -11.3 | 24 |
| Hippocampus | 7.09 | 7.13 | +0.56% | +0.29 | 93 |
| Inferior Lateral Ventricles | 0.89 | 1.02 | +15.9% | +8.3 | 57 |
| Thalamus | 10.57 | 10.27 | -2.83% | -1.48 | 9 |
| Cerebellum | 114.4 | 97.64 | -14.65% | -7.64 | NA |
| White Matter Hypointensity | 3.29 | 3.85 | +15.69% | +8.19 | 97 |
| EDSS | 4.5 | 4.5 |  |  |  |

**Table 9b. Patient 9.**

|  | April 2016  Volume  (cm³) | May 2019  Volume  (cm³) | 37 month  Percent  Change | Normative  Percentile for Patient’s Age | Normative  Percentile  For Patient’s  Age |
| --- | --- | --- | --- | --- | --- |
| Cortical Grey Matter | 421.4 | 434.9 | +3.2% | +1.04 | 47 |
| Superior Lateral Ventricles | 9.68 | 10.05 | +3.82% | +1.24 | 13 |
| Hippocampus | 7.04 | 7.21 | +3.0% | +0.97 | 95 |
| Inferior Lateral Ventricles | 0.61 | 0.60 | +1.67 | +0.54 | 15 |
| Thalamus | 10.5 | 11.2 | +6.67% | +2.16 | 52 |
| Cerebellum | 104.7 | 100.1 | -4.39% | -1.42 | NA |
| White Matter Hypointensity | 2.98 | 3.30 | +10.74% | +3.48 | 98 |
| EDSS | 1.5 | 2.0 |  |  |  |

**Table 10b. Patient 10**

|  | Dec 2014  Volume  (cm³) | Jan 2017  Volume  (cm³) | 37 month  Percent  Change | Normative  Percentile for Patient’s Age | 2017  Normative  Percentile  For Patient’s  Age |
| --- | --- | --- | --- | --- | --- |
| Cortical Grey Matter | 533.8 | 529.4 | -0.82% | -0.27 | 22 |
| Superior Lateral Ventricles | 20.0 | 21.87 | +8.9% | +2.89 | 70 |
| Hippocampus | 8.71 | 8.52 | -2.18% | -0.71 | 69 |
| Inferior Lateral Ventricles | 1.51 | 0.80 | -47.0% | -15.24 | 25 |
| Thalamus | 17.35 | 13.79 | -20.5% | -6.65 | 35 |
| Cerebellum | 147.5 | 145.6 | -1.3% | -0.42 | NA |
| White Matter Hypointensity | NA | 0.28 |  |  | 50 |
| EDSS | 2.0 | 2.0 |  |  |  |

**Table 11b. Patient 11.**

|  | Nov 2014  Volume  (cm³) | Sept 2018  Volume  (cm³) | 37 month  Percent  Change | Normative  Percentile for Patient’s Age | 2018 Normative  Percentile  For Patient’s  Age |
| --- | --- | --- | --- | --- | --- |
| Cortical Grey Matter | 699.73 | 688.1 | -1.66% | -0.54 | 57 |
| Superior Lateral Ventricles | 50.57 | 57.49 | +13.68% | +4.44 | 96 |
| Hippocampus | 9.86 | 9.34 | -5.27% | -1.71 | 30 |
| Inferior Lateral Ventricles | 1.24 | 1.50 | +20.97% | +6.80 | 47 |
| Thalamus | 19.4 | 18.9 | -2.58% | -0.84 | 76 |
| Cerebellum | 185.6 | 177.9 | -4.15% | -1.35 | NA |
| White Matter Hypointensity | 1.44 | 1.22 | -15.28% | -4.96 | 83 |
| EDSS | 2.5 | 3.0 |  |  |  |

**Table 12b. Patient 12**

|  | Dec 2019  Volume  (cm³) | Aug 2020  Volume  (cm³) | 9 month  Percent  Change | Normative  Percentile for Patient’s Age | 2020 Normative  Percentile  For Patient’s  Age |
| --- | --- | --- | --- | --- | --- |
| Cortical Grey Matter | 417.8 | 409.1 | -2.08% | -2.77 | 14 |
| Superior Lateral Ventricles | 21.18 | 22.52 | +6.33% | +8.44 | 55 |
| Hippocampus | 6.77 | 6.65 | -1.77% | -2.36 | 67 |
| Inferior Lateral Ventricles | 1.35 | 1.54 | +14.1% | +18.8 | 79 |
| Thalamus | 14.42 | 14.21 | -1.46% | -1.95 | 99 |
| Cerebellum | 128.0 | 134.3 | +4.9% | +6.53 | NA |
| White Matter Hypointensity | 1.19 | 1.26 | +5.88 % | +7.84 | 43 |
| EDSS | 1.5 | 1.5 |  |  |  |
